# Vision-based deformation recovery for intraoperative force estimation of tool–tissue interaction for neurosurgery

**DOI:** 10.1007/s11548-016-1361-z

**Published:** 2016-03-23

**Authors:** Stamatia Giannarou, Menglong Ye, Gauthier Gras, Konrad Leibrandt, Hani J. Marcus, Guang-Zhong Yang

**Affiliations:** The Hamlyn Centre for Robotic Surgery, Imperial College London, London, SW7 2AZ UK

**Keywords:** 3D stereo reconstruction, Soft tissue tracking, Deformation recovery, Force estimation

## Abstract

**Purpose:**

In microsurgery, accurate recovery of the deformation of the surgical environment is important for mitigating the risk of inadvertent tissue damage and avoiding instrument maneuvers that may cause injury. The analysis of intraoperative microscopic data can allow the estimation of tissue deformation and provide to the surgeon useful feedback on the instrument forces exerted on the tissue. In practice, vision-based recovery of tissue deformation during tool–tissue interaction can be challenging due to tissue elasticity and unpredictable motion.

**Methods:**

The aim of this work is to propose an approach for deformation recovery based on quasi-dense 3D stereo reconstruction. The proposed framework incorporates a new stereo correspondence method for estimating the underlying 3D structure. Probabilistic tracking and surface mapping are used to estimate 3D point correspondences across time and recover localized tissue deformations in the surgical site.

**Results:**

We demonstrate the application of this method to estimating forces exerted on tissue surfaces. A clinically relevant experimental setup was used to validate the proposed framework on phantom data. The quantitative and qualitative performance evaluation results show that the proposed 3D stereo reconstruction and deformation recovery methods achieve submillimeter accuracy. The force–displacement model also provides accurate estimates of the exerted forces.

**Conclusions:**

A novel approach for tissue deformation recovery has been proposed based on reliable quasi-dense stereo correspondences. The proposed framework does not rely on additional equipment, allowing seamless integration with the existing surgical workflow. The performance evaluation analysis shows the potential clinical value of the technique.

## Introduction

Microsurgical techniques are arguably the most technically demanding skills that trainee surgeons must learn. Mastery of microsurgery entails exceptionally delicate and precise tissue manipulation. In microneurosurgery, for example, sharp arachnoid dissection typically utilizes forces below 0.3N, levels that are often barely perceptible to surgeons. Failure to respect the force thresholds of fragile neurovascular tissue can result in iatrogenic injury, which in turn carries a risk of disability or even death. The clinical corollary is that tools that allow surgeons to better appreciate the forces they exert are likely to reduce the risk of operative complications, and therefore improve patient outcomes.

Thus far, research on the forces exerted during microsurgery has generally focused on the development of improved force feedback capabilities. Technological advances in surgical devices (NeuroArm, Steady-Hand Robot) and handheld instruments have allowed the integration of force sensing capabilities into surgical tools, resulting in the possibility of force feedback during an operation. However, the translation of these devices from the laboratory to the operating room is challenging due to the size of surgical instruments as well as sterility and biocompatibility issues [9]. As an alternative, vision can provide an indication of exerted forces. With experience, surgeons are able to rely on visual cues from the operative scene to estimate the forces they exert on delicate tissues, even in the absence of kinesthetic feedback. Hence, force estimation with direct vision-based approaches which are based on the use of the existing microscopic cameras has an advantage in that it allows seamless integration into the existing surgical workflow without the introduction of additional equipment to what is often an already crowded operating room.

Vision-based approaches for modeling tissue motion due to cardiac and respiratory cycles have used quasi-periodic or periodic signals to represent the tissue motion [11, 14]. However, recovering free-form tissue deformation due to tool–tissue interaction is more challenging. In [5], inertial sensors were used to increase the accuracy in the estimation of tissue displacements with monocular endoscopic cameras. In computer vision, template-based approaches have been proposed to reconstruct deforming structures assuming that the environment can be modeled as an isometric developable surface [15]. Since this is not a realistic assumption for soft tissue surfaces, quasi-conformal tissue surface modeling has been used in [1] for applications in MIS. The main disadvantage of the above monocular methods is the assumption that an initial 3D structure template is available or can be estimated. Also, approaches based on finite element and biomechanical models have been proposed to model tissue motion and estimate internal forces based on known preoperative displacements [12]. Stereo reconstruction has been used to recover the shape of deforming organs such as the heart [2, 8, 16]. However, none of these approaches have considered explicitly the recovery of tissue deformation across time.

Studies conducted in [10] have attempted to model surface tissue deformation using finite element simulations. The relationship between forces applied and tissue displacement that stems from [10] has been further analyzed in [6], to evaluate the propagation of displacements at different depths within the brain. This allowed for real-time estimation of displacements within the brain from forces applied on the tissue surface. These results demonstrate the possibility of deriving a relationship between the displacement of brain tissue and the forces exerted on that tissue.

The aim of this paper is to propose a vision-based approach for tissue deformation recovery. Accurate stereo correspondences are estimated in a novel fashion based on geometrical and appearance characteristics of salient features which provides quasi-dense 3D meshes. Probabilistic soft tissue tracking and thin-plate splines (TPS) are used to map 3D points of tissue surfaces over time and recover deformation. The extracted 3D displacements are used to estimate forces exerted on the tissue surface based on biomechanical modeling. Detailed validation with phantom data is performed, and the results derived justify the potential clinical value of the technique.

## Methods

In this study, stereo reconstruction is applied to data from calibrated cameras, with known intrinsic and extrinsic parameters. The epipolar constraint is imposed via image rectification to limit the search space for stereo correspondence.

### Stereo reconstruction

For reliable feature matching, the real-time affine-invariant anisotropic region detector [4] is employed since it is tailored for MIS applications and has high repeatability in challenging MIS data. Each salient point is characterized by its location $\mathbf x =(x,y)$, the saliency strength $c(\mathbf x )$, the parameters of the ellipse that represents the affine-invariant anisotropic region of the feature and the image region included into the ellipse. Given the small baseline between the laparoscopic cameras, corresponding features between the stereo frames are characterized by affine regions of similar shape. Also, corresponding regions on the left and right camera frames should represent the same image area and therefore should be described by similar intensities. Hence, the relative amount of overlap in the image covered by the regions to be compared and the dissimilarity in $c(\mathbf x )$ can be combined with appearance similarity and used as an indication of feature correspondence. Mathematically, this is defined as:

1 $$\begin{aligned} M_{i,j}=\frac{R_i\cap R_j}{R_i\cup R_j} + \frac{\min \left( c_i,c_j\right) }{ \max \left( c_i,c_j\right) } \end{aligned}$$

$R_i$ and $R_j$ represent the elliptic region of feature *i* on the left camera frame and feature *j* on the right camera frame, respectively. The saliency strength $c_i$ and $c_j$ is defined in a similar way, whereas $\cap$ is the intersection of the compared regions and $\cup$ is their union.

To establish a correspondence for feature *i* on the left camera frame, the similarity measure $M_{i,j}$ is calculated for all the features *j* on the right image that lie on the epipolar line and their region overlaps with *i* more than $60\,\%$ and the similarity of their image regions is high. A candidate correspondence for feature *i* is the feature with the highest similarity:

2 $$\begin{aligned} C&=\displaystyle \max _{j\in J} M(i,j) \nonumber \\ s.t.&\forall j: \frac{R_i\cap R_j}{R_i\cup R_j}>{\text{ thr }}_r \text { and } A_{i,j}>{\text{ thr }}_s \end{aligned}$$

where *J* denotes the search space defined by the epipolar line. $A_{i,j}$ represents the appearance similarity of the image areas covered by the compared ellipses, and normalized cross-correlation (NCC) has been used for that purpose. To deal with illumination variations between the stereo cameras, histogram equalization is applied to the compared image regions prior to similarity estimation. The threshold for the overlap score that defines the region correspondences is set to ${\text{ thr }}_r=60\,\%$ since it can guarantee successful region matching [4]. To reject false matches, the threshold for the appearance similarity of corresponding regions was set to a high value which in this study was empirically set to $90\,\%$.

For accurate stereo matching, a sparse set of reliable feature correspondences has been estimated. To enable a more detailed description of the observed surfaces, the density of feature correspondences is increased applying a local search for feature matches around the initial correspondences. Affine-invariant anisotropic features are detected in the areas defined by the initial matched ellipses, and correspondences are estimated as defined in Eq. (2).

To further increase the density of the stereo matches, feature propagation is proposed based on the affine consistency of the anisotropic regions [3]. That means if two neighboring features are located on a smooth and locally planar surface, the ellipses that describe the features will undergo similar affine transformations when the viewpoint changes. This is also a valid assumption for deformed surfaces as the affine transform varies smoothly within the surface.

Using the correspondences extracted from the global and local matching as seeds, affine-invariant anisotropic regions are propagated from the left to the right camera plane. The transformation between the regions $R^i_\mathrm{L}$ and $R^i_\mathrm{R}$ of two seed features $f^i_\mathrm{L}$, $f^i_R$ on the left and right camera plane, respectively, is defined as:

3 $$\begin{aligned} R^i_\mathrm{R}-f^i_\mathrm{R}=M^{-1}_\mathrm{R}RM_\mathrm{L}(R^i_\mathrm{L}-f^i_\mathrm{L}) \end{aligned}$$

where $M_\mathrm{L}$ and $M_\mathrm{R}$ are the affine transforms that normalize the ellipses into unit circles and *R* is the relative orientation between the regions. Hence, the correspondence of a candidate feature $f^c_\mathrm{L}$ to the right camera plane will lie at:

4 $$\begin{aligned} f^c_\mathrm{R}=M^{-1}_\mathrm{R}RM_\mathrm{L}f^c_\mathrm{L}+(f^i_\mathrm{L}-M^{-1}_\mathrm{R}RM_\mathrm{L} f^i_\mathrm{L}) \end{aligned}$$

**Fig. 1:**
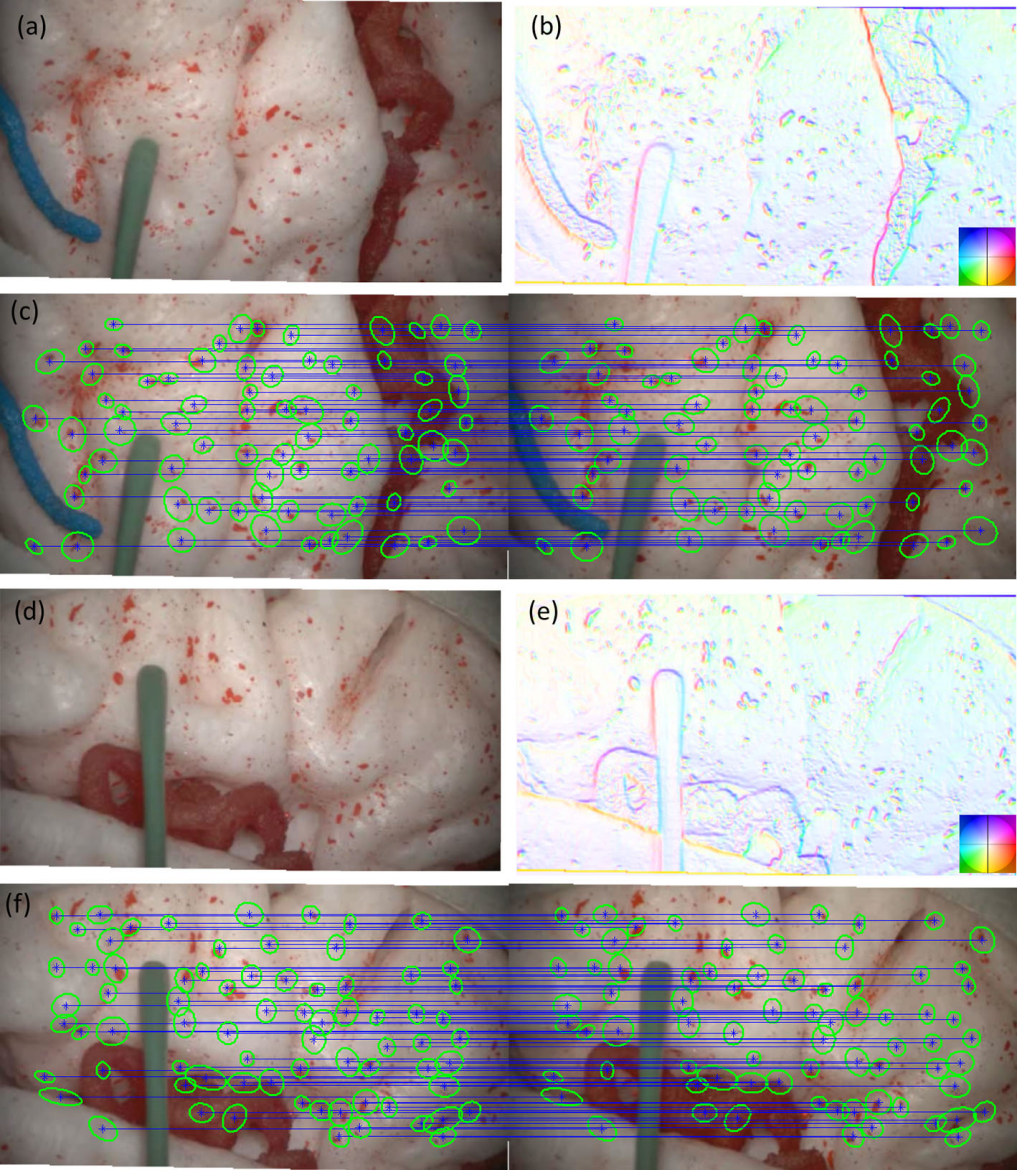
Example results of the proposed stereo matching approach. **a**, **d** Left camera images obtained from a stereo microscope; **b**, **e** estimated surface normals maps; **c**, **f** sample region correspondences

If the appearance similarity of the image regions around $f^c_\mathrm{L}$ and $f^c_\mathrm{R}$ is low, the matching is rejected as an outlier. The proposed correspondence propagation method is applied to a candidate feature if it lies within the area of a seed feature and their normals differ less than 1deg. Surface normals are defined as $N^{pq}=\frac{1}{\sqrt{p^2+q^2+1}}[p,q,-1]$, using the surface directional gradient vectors (*p*, *q*) estimated by approximating the image irradiance equation and the geometrical constraints of the endoscope camera and light sources [13].

Having selected reliable correspondences between the frames of the stereo cameras, a quasi-dense 3D point cloud *S* is estimated by triangulating the matched features using the camera extrinsic parameters. Samples of matched regions for pairs of stereo images are presented in Fig. 1, together with the maps of surface normals which have been used for stereo matching propagation.

**Fig. 2:**
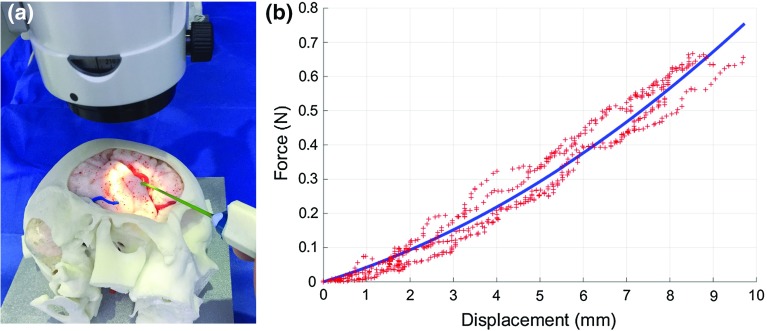
**a** The experimental setup which includes a microscope, a blunt surgical dissector and a realistic brain phantom. **b** The relationship between exerted force and tissue displacement

**Fig. 3:**
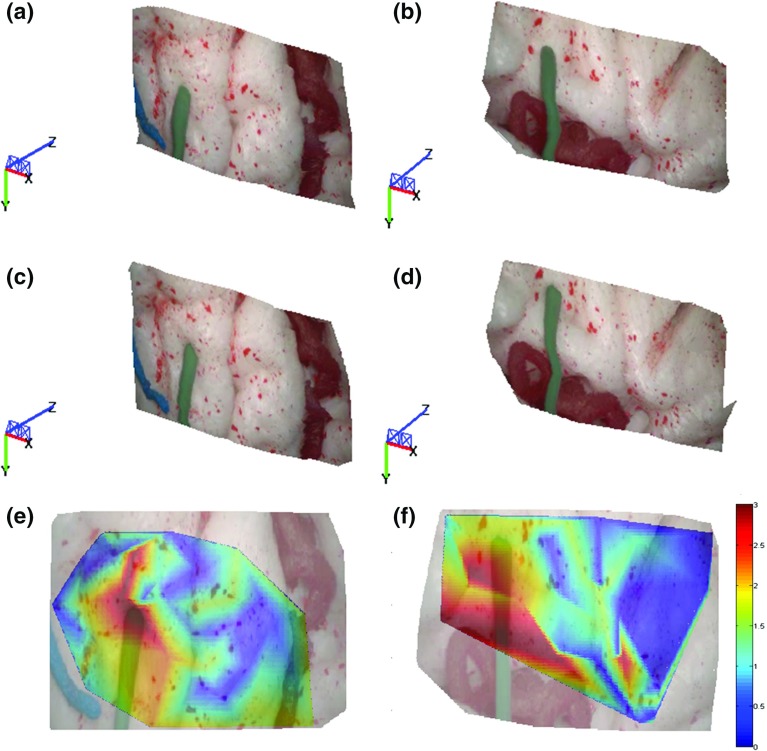
Sample results of the proposed framework. **a**, **b** Estimated 3D structure before tool–tissue interaction; **c**, **d** estimated 3D structure during tool–tissue interaction which causes tissue deformation; **e**, **f** deformation heatmaps overlapped on the images. The colormap units are in mm

**Fig. 4:**
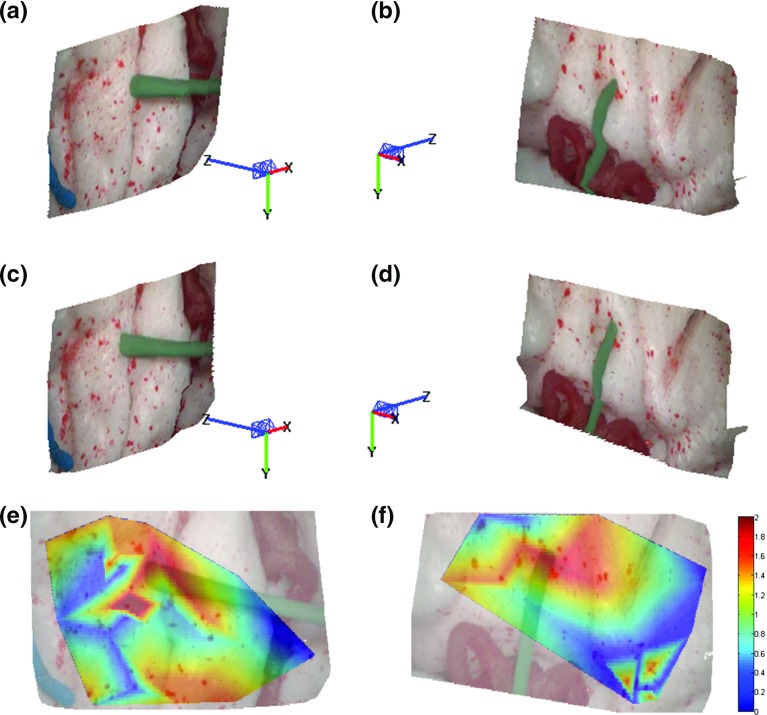
Sample results of the proposed framework. **a**, **b** Estimated 3D structure before tool–tissue interaction; **c**, **d** estimated 3D structure during tool–tissue interaction which causes tissue deformation; **e**, **f** deformation heatmaps overlapped on the images. The colormap units are in mm

### Tissue deformation recovery

In this work, we cast the problem of tissue deformation recovery as surface tracking over time. An implicit 3D tracking approach is proposed for that purpose. The extracted stereo correspondences are tracked on the image plane of the left camera with the probabilistic tracking approach proposed in [4]. At time *t*, if a feature ${f^t_\mathrm{L}}_i$ has been successfully tracked and reconstructed as $S^t_i$, it is used to estimate tissue deformation at this position at time *t* as $D({f^t_\mathrm{L}}_i)=\Vert S^t_i-S^O_i\Vert$, where $S^O$ is the tissue structure at a reference time instant.

However, feature matches extracted during the local matching and stereo propagation stages might not correspond to distinctive tissue landmarks and their spatiotemporal tracking might be unreliable. As a result, although 3D information is available for these features, their 3D displacement cannot be estimated over time. In order to recover deformation at as many points in *S* as possible, surface warping based on 3D thin-plate splines (TPS) is used to map an unstable 3D point $S^t_i$ to surface $S^{t+1}$. The 3D positions of the stable features $f_\mathrm{L}$ are used as control points. A point $S_i^t$ is mapped to $S_i^{t+1}$ which is then projected on the image plane $I^{t+1}_\mathrm{L}$ at position $w_i^{t+1}=K_\mathrm{L}P_i^{t+1}$, where $K_\mathrm{L}$ are the intrinsic parameters of the left camera. If 3D information $S_i^{t+1}$ is already available at $w_i^{t+1}$, then the tissue deformation at that point is recovered as $D(w_i^{t+1})=\Vert S^{t+1}_i-S^O_i\Vert$.

The estimated 3D displacements $D^{t}$ are used to estimate forces exerted on the tissue intraoperatively.

**Fig. 5:**
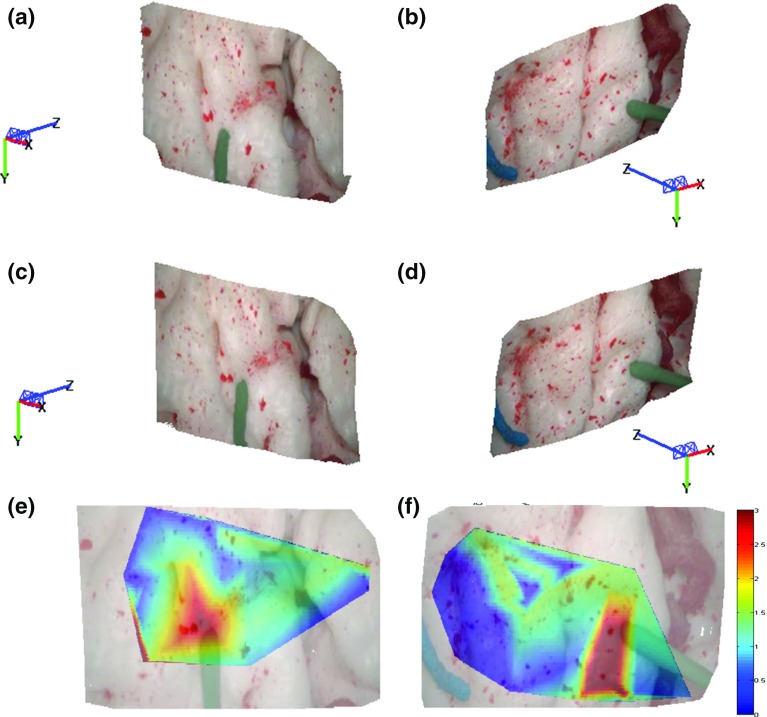
Sample results of the proposed framework. **a**, **b** Estimated 3D structure before tool–tissue interaction; **c**, **d** estimated 3D structure during tool–tissue interaction which causes tissue deformation; **e**, **f** deformation heatmaps overlapped on the images. The colormap units are in mm

### Force estimation

For intraoperative force estimation using visual cues, a force–displacement model needs to be generated to establish a relationship between tissue displacement and exerted forces. In this paper, a method similar to the one presented in [10] was used to characterize the material properties of the brain phantom used for the experiments. A force sensor was used to record forces applied on the tissue, while electromagnetic (EM) tracking was used to record the displacement on the tissue surface introduced by these forces. A downward force exerted from the top of the brain tissue simulated the push of a blunt dissector. Force and displacement measurements were collected for different areas of the model surface as shown in Figs. 3,  4 and 5. The measured force–displacement graph of the combined data is shown in Fig. 2b. As it can be seen in this figure, different tissue areas yielded similar force–displacement relationships, which are also similar to the experimental force–displacement curve estimated in [10] using in vivo porcine data. A force–displacement model was created using a second-order polynomial fitting, which was found to provide the best fit to the combined data. The force–displacement model is shown as the solid line in Fig. 2b.

In order to evaluate the force applied by the tool on the tissue, the surface displacement that the tool generates at the point of contact must be recovered. However, it is difficult to estimate the displacement directly using a visual method, as the tool is occluding the image area at that point. In this work, the displacement at the tool–tissue contact point is inferred through a model by taking advantage of the known movement of the features in the vicinity of the tool tip. A curve-fitting approach was used to model the 3D tissue deformation as a Gaussian distribution. The movement of features around the tool tip is tracked using the probabilistic approach in [4] and is compared to their position prior to the tool–tissue interaction. The resulting 3D displacements are used to quantify the parameters of the Gaussian distribution. The tissue displacement at the tool tip corresponds to the peak of the Gaussian distribution. Finally, the force is recovered using the relationship established between surface displacement and force, presented in Fig. 2b.

## Experimental results

In order to assess the accuracy of the proposed framework on a clinically relevant experimental setup, a brain modeled anatomical replica (Kezlex, Tokyo, Japan) shown in Fig. 2a was used. The model is made from soft elastomer and provides a feel similar to the real brain. The realistic material properties of the used brain model can also be verified by the estimated force–displacement graph in Fig. 2b which is similar to the curve estimated in [10] using in vivo porcine data. The validity of the Kezlex model has also been studied in [7] on simulated surgical tasks, including arachnoid dissection.

For quantitative and qualitative performance evaluation stereoscopic video data were collected with a Leica M525 OH4 (Leica Microsystems Ltd., UK) surgical microscope. For tissue manipulation, a blunt surgical dissector (Yasargil FD304R, Braun Aesculap) axially affixed to a 6 degree-of-freedom force/torque sensor (ATI Nano17, ATI Industrial Automation, Inc) that can sense both lateral and axial forces applied to the dissector tip. The handheld tool was tracked with 6 degree-of-freedom EM sensor (3D Guidance trakSTAR, Ascension Technology Corporation).

**Fig. 6:**
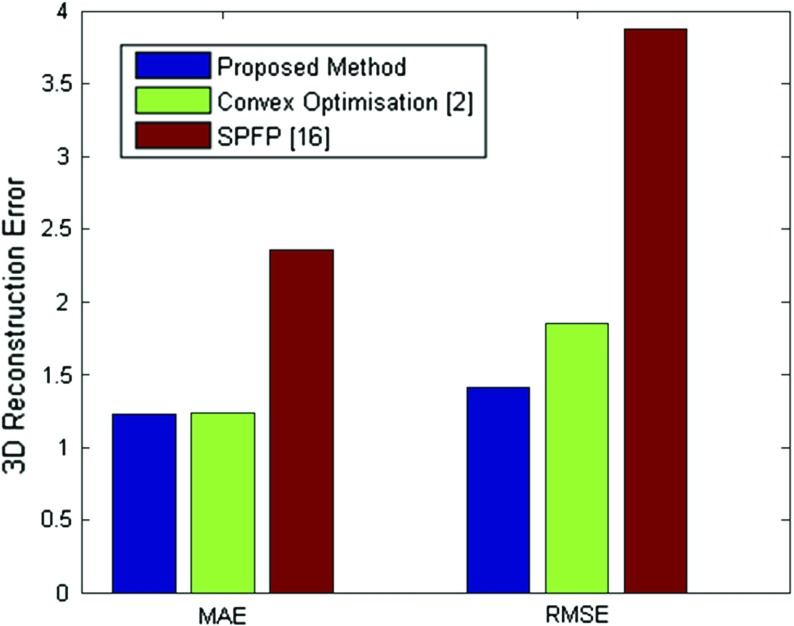
The comparison results of the proposed stereo reconstruction and the state-of-the-art methods

The force sensor and the EM tracking served two roles in our experiments. The first was to provide a ground truth during the evaluation of forces and displacements of the tissue. The second was to perform an initial modeling phase to quantify the material properties of the tissue of the brain phantom. This was performed prior to the validation experiments and was used to establish a relationship between tissue displacement and forces exerted. The results, displayed in Fig. 2b, are comparable to those reported by [10].

The generation of ground truth data for the 3D structure of the surgical environment during intraoperative tool–tissue interaction is not always feasible, and standard techniques involving the use of optical tracking devices and computerized tomography (CT) scanners are not applicable. In this work, the ground truth data were obtained manually with detailed annotation by an experienced observer. A sample of the extracted stereo correspondences is examined along time, and their position is selected on both camera planes in order to estimate ground truth 3D positions and hence ground truth deformations.

**Table 1:** Performance evaluation on brain phantom data

Error	3D reconstruction error (mm)		Deformation error (mm)	Force estimation error (N)
	Mean	SD	Mean	Mean
Seq. 1	0.87	0.55	0.22	0.07
Seq. 2	0.57	0.45	0.05	0.03
Seq. 3	0.62	0.39	0.12	0.05
Seq. 4	0.39	0.33	0.18	0.12
Seq. 5	0.44	0.43	0.02	0.01
Seq. 6	0.54	0.39	0.17	0.04

To validate the proposed stereo matching approach, we have used the cardiac phantom data from the Hamlyn surgical vision dataset where ground truth is available [4]. We have compared our method to state-of-the-art dense and semi-dense approaches including [2] and [16], respectively. It can be seen from Fig. 6 that our approach provides the highest accuracy, outperforming the compared methods.

To further validate the proposed framework, we carried out a performance evaluation study on a brain phantom with generated tissue deformations up to 10mm. The ground truth data were manually labeled as explained above. The 3D reconstruction accuracy of the proposed method on the brain phantom data is provided in Table 1 which shows that the method achieves submillimeter reconstruction accuracy. The accuracy of the estimated forces measured on the phantom using the recovered 3D point displacements is also presented in Table 1. The relatively high force error in Sequence 4 is due to a slight sensor biasing error introduced during that measurement.

Qualitative results of the performance of the proposed method are shown in Figs. 3, 4 and 5 where the 3D structure of the observed environment and the heatmap of the estimated deformation in the surgical scene are presented for different levels of deformation. The 3D structures in Figs. 3,  4 and  5a–d were generated applying the proposed quasi-dense stereo reconstruction method. The smoothness in the recovered surfaces shows the ability of the method to reject outliers. The heatmaps in Figs. 3, 4 and 5e, f show that high levels of deformation have been recovered in areas affected by the tool–phantom interaction, while in static areas the recovered deformation was low. Outliers have been detected in small textureless areas (Fig.  6).

## Conclusions

In this paper, a novel approach for tissue deformation recovery has been proposed based on reliable quasi-dense stereo correspondences. Probabilistic tracking and surface mapping have been used to estimate 3D displacements along time. The proposed approach can recover free-form tissue deformation without making any assumptions about the motion or the physical properties of the tissue. The accuracy of the method and its robustness to the degree of exerted forces has been shown, and the application of this method to estimating tissue forces has been demonstrated. The proposed framework does not rely on additional equipment, allowing seamless integration with the existing surgical workflow. Quantitative and qualitative performance evaluation shows the potential clinical value of the proposed framework. The method currently is not real time, but the algorithm can be easily parallelized and implemented using GPGPU.
